# Integration of laboratory and process testing data

**DOI:** 10.1155/S146392469500023X

**Published:** 1995

**Authors:** Michael Tyszkiewicz

**Affiliations:** Automated Compliance Systems Inc. 245 Highway 22 West Bridgewater New Jersey 08807 USA

## Abstract

The author describes ACS Inc.'s Pro-LIMS system which integrates laboratory and process procedures. The system has been shown to be an important toolfor quality assurance in the process manufacturing industry.


Journal of Automatic Chemistry, Vol. 17, No. 4 (July–August 1995), pp. 161–162

### Integration of laboratory and process testing data

**Michael Tyszkiewicz**

*Automated Compliance Systems, Inc., 245 Highway 22 West, Bridgewater, New Jersey 08807, USA*

*The author describes ACS Inc.'s Pro-LIMS system which integrates laboratory and process procedures. The system has been shown to be an important tool for quality assurance in the process manufacturing industry.*

The process industry is undergoing radical changes because of customer-driven requirements for lower prices and higher quality. The 1980s brought about quality initiative, continuous improvement, and ISO 9000 programmes that focused on industry practices and methods. Process automation systems focused strictly on process variables and process control, while Laboratory Information Management Systems (LIMS) traditionally focused on samples and data strictly from the laboratory perspective. Functionality was developed in both of these systems to meet end-user workflow requirements and increase efficiency. The linking of laboratory data to the process area—which is required to make instantaneous process decisions—has not been widely addressed.

### Current methods for integration of data

While relational database products such as Oracle have made the exchange of information much easier, current LIMS technology falls short of total process integration. LIMS packages use *status changes* of samples as triggers to send messages and data to other systems. In order to track data, samples and tests are assigned a status once they arrive in a laboratory. Some of the common status descriptors are pre-logged, logged (received in the laboratory), completed and authorized. LIMS vendors then write routines that trigger site-specific functional reports or actions at status changes. These actions range from simple tasks like sending data in file form for another system to read at sample completion, to complex procedures such as running product-grading routines. This strategy assumes that all product quality data is owned and maintained by the laboratory. However, this assumption only holds true at a small number of installations.

### Sample statuses and their limitations

The total integration of a process laboratory with the processing area involves more than just the exchange of in-lab data in a timely fashion. In an area where several process steps are involved in production, there are many interactions between the laboratory and process area, and many human tasks and decisions affect product quality and operational efficiency. Most of these take place without computer assistance or electronic documentation. Simple visual inspections of product, or physical and visual testing performed in the process area, are critical data points that result in approval to continue the next process step. The laboratory and process area must be integrated to work as one unit to achieve high product quality and maximum efficiency. This will only be possible when process area data and procedures that affect product quality are integrated with in-lab data to allow statistical analysis of these variables for process optimization.

All LIMS systems have a limited number of status changes. If a user needs more statuses than supplied in the LIMS, the amount of customization necessary becomes staggering. This phase of the implementation can sometimes become the most time-consuming and costly part of a project. In many cases, product quality is determined at different production areas, or at intermediate process steps by either sophisticated equipment or visual inspections. The incorporation of process data points, on-line visual inspections, and manual approval steps into any system, has proven to be the most difficult phase of system implementation. The project team is faced with the dilemma of heavily customizing a LIMS to add many more status updates to accommodate all of these multiple steps, or modifying existing production procedures to allow the software to fit the application. Either approach is not justifiable, and in most cases the integration of this data is determined to be unfeasible.

### Examples from the process industry

One example of status limitations in the pharmaceutical industry is where several levels of approval, instead of one, would be necessary before sending data out of the laboratory. The major customization, maintenance, and effort required for the work-around may still leave the end-user only partially satisfied with the end results. Another good example is in the process manufacturing industry. A product being manufactured on a conveyor line may have hundreds of process steps and visual inspections at checkpoints. If any of the visual inspections are not performed, faulty materials or parts may be installed on the finished product, requiring costly production slowdowns, or product recalls. Handling this scenario with current LIMS offerings is difficult, since two systems must be in constant communication with each other through customized links, and too many process area data points are omitted from on-line analysis. The real-time link from process to the quality control area is incomplete or interrupted, thereby creating loopholes in the quality assurance process.

Automated Compliance Systems Inc. (ACS) has analysed the integration requirements and status limitations and

0142-0453/95 $10.00 © 1995 Taylor & Francis Ltd.

designed Pro-LIMS, which is a LIMS that addresses the needs of the process industry.

### *Pro-LIMS event/action builder*

Pro-LIMS was built around the theory that sampling, data entry, and reporting are events that occur on a conveyor belt. Pro-LIMS allows users to configure their system with an unlimited number of user-defined events and actions. Sample pulls can be scheduled on a calendar basis or attached to processes and process steps in a Microsoft Windows Graphical User Interface (GUI). Since processes can be broken down into an unlimited number of process steps, the end user can easily schedule and link laboratory events based upon plant actions and vice versa. The linkage of process steps to events allows easier and better definition of multi-phase batch operations. The end user can also define actions and events based upon shift schedules, for better definition and management of continuous operations. All of the pre-logged events can then be viewed in the forecasting utility, allowing the user an approval step of the automatically scheduled work with a chance to cancel some or all of it, if desired.

### *Pro-LIMS robot chains*

Another advantage of Pro-LIMS is robot chains. Robot chains are used instead of sample and test statuses to handle data flow. ACS offers an unlimited number of configurable user-defined steps that follow existing standard operating procedures to trigger and enforce required actions. A robot chain can include any number of fully computerized, computer-assisted, and human actions that force compliance to procedures and/or force the order of events. The human actions may be visual inspections, product-preparation steps, or process testing, which occurs in the process area, that can now be integrated, enforced and electronically documented. The pharmaceutical industry's needs are met with simple configuration of a robot chain that includes extra levels of approval before data is transmitted to process areas. The robot chain monitor gives the end-user a tool to easily view the in-process and finished items, so corrective actions may begin. The transmission of the data is identified as a process step in the chain. A process manufacturing system that needs to monitor visual inspections of product will find robot chains to be the perfect solution. Since robot chains can force the order of events, the visual check and approval at a particular process step will immediately cause disruption of the chain if it is not completed. In this case, a background process monitors elapsed times and notifies someone to take action. No quality assurance-driven events can take place until the approval is given and electronically documented, thereby preventing a product from being released from QC hold until an inspection in the process area takes place.

### *Pro-LIMS benefits for process solutions*

Compliance is a major issue in all industries. The issue is being driven by ISO 9000 in process manufacturing, the FDA in pharmaceuticals, as well as other quality assurance programs. Adherence to compliance is a totally procedure-driven effort. Most of these procedures are manual, making it extremely difficult or impossible for current LIMS technology to add any value in this effort. Pro-LIMS robot chains provide a tool to configure a system to include all laboratory and process procedures, while enforcing order and compliance to them. The extension of these capabilities from the laboratory into the process area totally integrates the two separate areas, which provides an important tool for quality assurance.

